# Recent Trends in Controlling the Enzymatic Browning of Fruit and Vegetable Products

**DOI:** 10.3390/molecules25122754

**Published:** 2020-06-15

**Authors:** Kyoung Mi Moon, Eun-Bin Kwon, Bonggi Lee, Choon Young Kim

**Affiliations:** 1 College of Pharmacy and Research Institute of Pharmaceutical Sciences, Gyeongsang National University, Jinju 660-701, Korea; omkksm@nate.com; 2 Korean Medicine (KM) Application Center, Korea Institute of Oriental Medicine (KIOM), Dong-gu, Deagu 701-300, Korea; wrld2931@kiom.re.kr; 3 Department of Food Science and Nutrition, Pukyong National University, Nam-gu, Daeyeon Dong, Busan 608737, Korea; 4 Department of Food and Nutrition, Yeungnam University, Gyeongsan, Gyeongbuk 38541, Korea

**Keywords:** natural anti-browning agents, polyphenol oxidase, PPO inhibitor, sustainability, food waste utilization, nutritional values

## Abstract

Enzymatic browning because of polyphenol oxidases (PPOs) contributes to the color quality of fruit and vegetable (FV) products. Physical and chemical methods have been developed to inhibit the activity of PPOs, and several synthetic chemical compounds are commonly being used as PPO inhibitors in FV products. Recently, there has been an emphasis on consumer-oriented innovations in the food industry. Consumers tend to urge the use of natural and environment-friendly PPO inhibitors. The purpose of this review is to summarize the mechanisms underlying the anti-browning action of chemical PPO inhibitors and current trends in the research on these inhibitors. Based on their mechanisms of action, chemical inhibitors can be categorized as antioxidants, reducing agents, chelating agents, acidulants, and/or mixed-type PPO inhibitors. Here, we focused on the food ingredients, dietary components, food by-products, and waste associated with anti-browning activity.

## 1. Introduction

### 1.1. Enzymatic Browning

Browning is a process of gradual change in the color of food products to brown or dark brown over time, which can affect the food quality in either a positive or negative manner [1]. This reaction is considered undesirable for most fruit and vegetable (FV) products and seafood such as shrimp; however, it is important to produce a unique color and flavor in some other foods, such as bread, soy sauce, black tea, coffee, cocoa, raisins, and dried jujube [2,3]. The browning reaction in food products is generally divided into enzymatic and non-enzymatic browning, depending on the mechanism. The non-enzymatic browning reaction generates a brown-colored substance through a chemical reaction involving a single compound or multiple constituents in food, without having any enzyme involved. The non-enzymatic browning reactions include the Maillard reaction, caramelization, and ascorbic acid oxidation; in food products, these reactions occur mostly in a combination rather than as isolated reactions, because food is composed of complex constituents [4]. Unlike non-enzymatic browning, the enzymatic browning reaction involves the action of the polyphenol oxidase (PPO) enzyme present in food. Enzymatic browning mostly occurs in FV products during harvesting, transportation, storage, and processing; consequently, it influences the sensory and nutritional values of food products [5]. Mechanical and physical stimuli involving peeling, cutting, slicing, dicing, and shredding during food processing and severe temperature changes during storage can cause physical tissue damages in FVs. Owing to tissue damage, the phenolic compounds and PPOs found in food are exposed to oxygen, initiating the oxidation of phenols into quinones. Subsequently, these quinones and their derivatives are polymerized through several reactions, forming a relatively insoluble brown pigment known as melanin [5]. The rate of enzymatic browning is determined by the enzymatic activity of PPOs.

### 1.2. Polyphenol Oxidase Activity and Its Prevention

PPO (EC1.10.3.1) is a copper-containing enzyme that belongs to the family of oxidoreductases, which are classified into EC1.14.18.1 (monophenol monooxygenase, cresolase, or tyrosinase) and EC1.10.3.1 (diphenol oxidase, catechol oxidase, or *o*-diphenol oxygen oxidoreductase) by the Enzyme Commission (EC) according to substrate specificity. Generally, PPO refers to an enzyme belonging to EC1.10.3.1. PPO is present in the tissues of plants and animals, especially fruits (apple, peach, pear, banana, apricot, berries, mango, avocado, and grape) and vegetables (potato, lettuces, burdock, guava, melon, eggplant, and mushroom) [1,6]. Because the PPO activity is crucial for controlling enzymatic browning, the factors influencing PPO activity, including the type and amount of endogenous phenol compounds, presence of oxygen, and pH are targeted to prevent enzymatic browning.

The approach for the prevention of enzymatic browning is divided into physical and chemical methods. Physical methods to regulate enzymatic browning include thermal treatment, prevention of oxygen exposure, use of low temperature, and irradiation. Heat treatment, such as blanching, can easily inhibit the enzymatic activity because enzymes, which are composed of proteins, are denatured [7,8]. In the wine-making process, enzymatic browning is suppressed by heat treatment at 60 °C for 3 min before brewing [9]. However, blanching could cause undesirable generation of color or flavor and softening of texture; thus, it is rarely used for frozen fruits consumed without cooking. When blanching cannot be used, the browning reaction can be suppressed by eliminating the oxygen. Substituting air with an inert gas (N_2_ and CO_2_), the use of oxygen-impermeable packaging films or edible coating, or immersion of foods in a sugar or salt solution of a certain concentration can be used to avoid contact with oxygen [10,11,12,13,14]. Moreover, lowering the temperature by precooling, refrigeration, and freezing, and using ultraviolet C and gamma-irradiation are potential techniques to suppress enzymatic browning [15,16].

**Figure 1:**
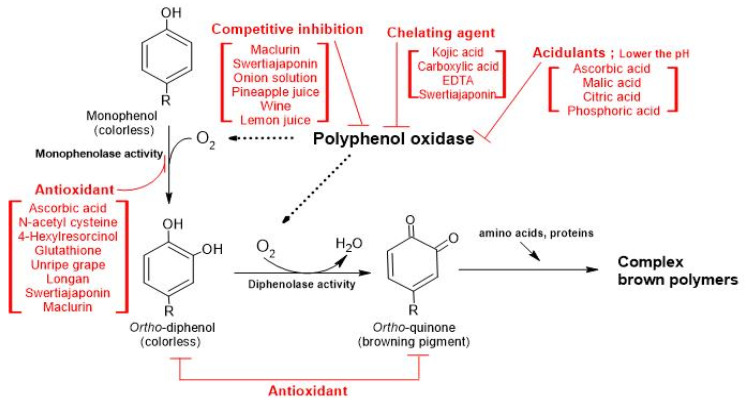
The simplified processes of enzymatic browning and inhibition mechanisms of anti-browning agents. The processes of enzymatic browning initiating with monophenolase activity from a para-phenolic compound to a 3,4-polyphenol followed by enzymatic polyphenol oxidase activities to produce the corresponding *ortho*-quinone derivative. Red letters indicate where the anti-browning agents possibly work on the processes of enzymatic browning. This figure was modified from Linda et al. [21]. EDTA; ethylenediaminetetraacetic acid.

Chemical methods to inhibit PPO activity include acidification or reduction using antioxidants, chelating agents, or natural extracts (Figure 1). PPO with an optimal activity at pH 5–7 shows inhibition below pH 3.0 [17]. Acidifying agents, such as citric acid, ascorbic acid, and glutathione can inactivate PPO by lowering the pH. The reducing agent, sulfate, and its derivatives act as irreversible inhibitors of PPO [18]. Antioxidant agents including ascorbic acid, l-cysteine, and 4-hexylresorcinol, are able to prevent melanin formation by binding to the intermediates [19]. Copper-chelating agents, such as citric and oxalic acids are able to suppress PPO activity by binding to metal cofactors in the PPO enzyme structure [20].

For the quality of FV products, different combination application of physical and chemical methods to inhibit browning reaction is more effective and research on this has been actively conducted. However, in order to find the development strategies of anti-browning methods, it is necessary to take into account the consumers’ needs for the selection of FV products.

### 1.3. Current Trends in the Development of Anti-Browning Agents

As the fresh-cut FV market is a large and rapidly growing segment in food processing industries [22], the challenge for food processors is the application of appropriate additives to control enzymatic browning for maintaining quality and extending the shelf life of fresh-cut products, while meeting the needs of current consumers. A growing number of consumers are demanding foods that are safer, healthier, and better for the environment.

Consumers are reluctant to buy products with synthetic additives and would prefer to replace those with natural ingredients because of their preference for safer and healthier foods. Synthetic additives often cause harmful effects on health. One of the extensively used PPO inhibitors, sulfite, was prohibited for use in FVs by the United States Food and Drug Administration in 1986 because of serious health issues [23]. Since enzymatic browning deteriorates the nutritional value of FV products because of the degradation of phenolic substrates by PPO, the inhibition of PPO itself ensures preserving the nutritional quality of FV. To satisfy the consumers, finding a natural PPO inhibitor with additional health benefits becomes an emerging interest. Plant extracts are known to contain high levels of phenolic substances, which provide the health benefits of FV consumption and also contribute to color, flavor, astringency, and bitterness [24]. Therefore, the utilization of plant extracts as PPO inhibitors is a suitable strategy to meet consumer needs while producing value-added FV products.

In addition, consumers have become more aware of sustainability, and thus, they are willing to spend more on sustainable food products [25]. Both the reduction of food waste and valorization of agro-food by-products and waste are important issues in food production. Since 50% of the fresh fruit loss is because of color deterioration caused by the PPO enzyme, proper control of browning may reduce the elevated economic loss and food wastage [3]. Thus, identification of a sustainable PPO inhibitor is one of the approaches to improve food sustainability. Agro-food by-products and wastes are an appropriate source for extracting bioactive components that can be used as ingredients for functional foods and food additives [26,27]. Moreover, studies focused on bioactive components isolated from agro-food by-products and waste have reported that these by-products possess both potent antioxidant activities and anti-browning properties [28]. Herein, we specifically focused on natural extracts as PPO inhibitors of FVs that are adequate to replace the synthetic additives while enhancing nutritional values. In addition, PPO inhibitors derived from agro-food by-products and waste have been reviewed.

## 2. Common Mechanisms Underlying the Anti-Browning Activity of Chemical PPO Inhibitors

### 2.1. Antioxidants/Reducing Agents

Antioxidants can react with oxygen to suppress the initiation of browning. They are also able to react with the intermediate products, thereby breaking the chain reaction and inhibiting melanin formation [29]. The anti-browning effects of antioxidants rely on environmental factors including temperature, pH, light, and composition of the atmosphere [30]. Ascorbic acid, *N*-acetyl cysteine (NAC), hexylresorcinol, erythorbic acid, cysteine hydrochloride, and glutathione are antioxidants that have been widely studied for preventing the browning of fruits (Table 1) [31,32,33].

Oms-Oliu et al. investigated the individual and combined anti-browning effects of NAC, reduced glutathione, ascorbic acid, and 4-hexylresorcinol on pear [32]. NAC at 0.75% efficiently blocked the browning of fresh-cut pears for up to 28 days at 4 °C. Reduced glutathione also suppressed the browning of pears over the storage time, and browning of pear dipped in 0.75% reduced glutathione was observed after 21 days of storage [32]. However, the treatment of 4-hexylresorcinol or ascorbic acids did not fully inhibit the browning of pears over the storage time. When both NAC and reduced glutathione were combined, a better anti-browning activity was achieved [32]. The combined effects of the anti-browning agents on apples have also been reported. A previous study indicated that 4-hexylresorcinol at >0.5%, *N*-acetylcysteine at <0.75%, and *N*-acetylcysteine combined with glutathione at <0.6% exhibited strong anti-browning activities in fresh-cut Fuji apples during 14 days of storage at 4 °C (Table 1) [40].

Ascorbic acid is widely used as an anti-browning agent. The mechanism underlying the anti-browning activity of ascorbic acid appears to rely on its reducing activity. Although ascorbic acid does not directly interact with PPO enzyme, it inhibits enzymatic browning by reducing oxidized substrates [33]. Other studies also suggested that the anti-browning function of ascorbic acid can be attributed to the reduction of enzymatically formed *o*-quinones to their precursor diphenols [34,35]. Nevertheless, the anti-browning property of ascorbic acid may not be strong when applied to fresh-cut pears. When ascorbic acid is fully oxidized to dehydroascorbic acid, *o*-quinones can no longer be reduced to diphenols and browning probably still occurs because of melanin generation [41].

### 2.2. Chelating Agents

Chelating substances are also widely used to inhibit PPO activity because they can form complexes with Cu(II) present in PPO or react with their substrates, thereby suppressing enzymatic browning [31]. Kojic acid is a fungal metabolite generated by several species of *Aspergillus* and *Penicillium*. It is a strong chelator of metal ions including Fe(III) and Cu(II) [42]. Therefore, the binding of kojic acid to Cu(II) in the PPO enzyme inactivates PPO. A previous study revealed that kojic acid inhibited enzymatic browning in apple slices, and its inhibitory effects were stronger than those of caffeic, ferulic, chlorogenic, coumaric, cinnamic, and gallic acids (Table 2) [43]. However, it is not commonly used in the food industry, possibly because of the difficult process involved in its large-scale production and high cost [31].

Reportedly, most of the carboxylic acids exhibit anti-browning activities because of their metal-chelating activities or pH-lowering effects [31,43,50]. Son et al. investigated the anti-browning effects of twelve carboxylic acids that are commonly found in FV. The browning extent of the apple slices treated with an individual carboxylic acid solution (1%) for 3 min, was monitored at room temperature for 3 h [43]. Tartaric, malonic, oxalic, and oxalacetic acids showed strong inhibition against the browning of apple slices; citric, lactic, malic, and pyruvic acids exhibited moderate inhibition, whereas weak inhibition was observed with fumaric, acetic, succinic, and formic acids [43]. Although the anti-browning activity of citric acid is not that strong when compared with other carboxylic acids, it has been widely used as an anti-browning compound in the food industry [51].

The wide application of citric acid attributes to its general recognition of safety, pleasant acid taste, high water solubility, and chelating and buffering properties [52]. Thus, citric acids applied to various foods and beverages including wines, ciders, soft drinks, syrups, jellies, dairy products, frozen fruits and candies for elevating tartness, natural fruit flavor, the effectiveness of antimicrobial preservatives and antioxidant capacity and for minimizing crystallization of sucrose and enzymatic browning [52].

Oxalic acid broadly exists as its potassium or calcium salt in nature. It has been reported that vegetables, including spinach, rhubarb, and beet root, contain approximately 356–780, 260–620, and 97–121 mg/100 g of oxalic acid, respectively [53]. Considering its widespread occurrence in nature and strong anti-browning activity, oxalic acid and its derivates have been used as anti-browning agents for fresh-cut apple slices [43]. Ethylenediaminetetraacetic acid (EDTA) and its sodium salt are also broadly applied as metal chelating compounds in the food industry. However, they are generally combined with other compounds including ascorbic acids and citric acids to suppress the browning of foods [34].

### 2.3. Acidulants

Acidulants, especially the naturally occurring ones in the tissues, are also widely used as anti-browning agents, and these include ascorbic, malic, citric, and phosphoric acids. Generally, PPO is active at pH 6–7, but inactive below pH 3. Acidulants lower the pH, and thereby decrease the enzymatic activity of PPO. The enzymatic browning of sugarcane juice was efficiently inhibited below pH 4.1 when it was treated with ascorbic or citric acid (Table 2) [54]. Even though tartaric, erythorbic, acetic, and malonic acids are also acidifying agents having anti-browning activity, they alone are rarely used to prevent the enzymatic browning of FVs. The acidifying agents are often treated with other anti-browning agents, such as antioxidants, chelating agents, and enzyme inhibitors [31,54].

### 2.4. Mixed-Type Inhibitors

Although the anti-browning agents are categorized based on their major functional characteristics, many compounds show multiple mechanisms for the anti-browning effects. The compound 4-hexylresorcinol plays a dual role in PPO enzyme. If there are no substrates, it interacts preferably with the deoxy form of PPO, decreasing PPO activity. However, if substrates are present, 4-hexylresorcinol competes for the catalytic site of PPO as a canonical enzyme inhibitor, subsequently binding to the met form of PPO [33]. Maclurin appears to have stronger antioxidant capacity than ascorbic acid. It may also bind to and inactivate PPO by forming multiple hydrogen bonds and aromatic interactions with the binding pocket based on the protein–ligand docking simulation followed by binding residue analysis (Table 3) [55]. Swertiajaponin appeared to have triple functions in inhibiting enzymatic browning (Table 3). It is an antioxidant and may bind and inactivate PPO by forming 4 hydrogen bonds and a hydrophobic interaction [56]. In addition, swertiajaponin showed copper chelating activity at <50 μM in a concentration-dependent manner (50–500 μM). Especially, the copper chelating activity of swertiajaponin at 500 μM was comparable to that of the positive control, EDTA at 3.4 mM [56]. Furthermore, many acidifying agents exert multiple effects to suppress enzymatic browning. For example, citric, malonic, and acetic acids possess both chelating and acidifying properties. Ascorbic acid is a strong antioxidant with acidifying characteristics.

Chemical anti-browning agents to inhibit PPO activity including antioxidants, reducing agents, chelating agents, acidulants, and mixed-type inhibitors are effective at controlling the browning of FV. Even though these conventional treatments are commonly utilized in FV products, degradation of sensory and nutritional aspects of FV products and concerns regarding health have prompted the search for a novel way to regulate PPO.

## 3. Natural Anti-Browning Agents

### 3.1. Onion

Onion is considered as a healthy food with numerous benefits as it contains various functional compounds, such as anthocyanins, kaempferol, quercetin, isorhamnetin, and alkyl cysteine sulphoxides [58]. Additionally, onion extracts have been reported to suppress the enzymatic browning of potato by inhibiting PPO activity [59]. Not only fresh onion extract but also the heated one can prevent the browning of potatoes. It seems that the heated onion extract is more potent than the fresh ones. The anti-browning effect of the onion extract on potato PPO was dependent on the heating temperature. Furthermore, the supplementation with glucose and glycine further elevated the suppressive effect of the onion extract against PPO [59]. In another study, the onion extracts suppressed the enzymatic browning of pear [60]. Water-based onion extract significantly inhibited the PPO activity of pear and the heated one further suppressed it. It appears that the heating temperature and time are positively associated with the extent of the inhibitory effect of the onion extract on the PPO activity [60]. In addition, heated onion extract ranged from 20 to 100 mg/mL exhibited a dose-dependent inhibitory effect on the pear browning. Consistently, both the fresh and heated onion extracts delayed the browning of pear juice. The mechanism of action of the onion extract seems to include but is not limited to non-competitive inhibition of pear PPO (Table 4) [60].

Another study further investigated the effects of the addition of onion on browning, nutritional value, and especially antioxidant characteristics of apple juice. The authors specifically compared the degree of browning and nutritional quality of fresh and heated apple juices pre-supplemented with onion [61]. The fresh apple juice supplemented with onion exhibited decreased browning and increased total phenolic compound content, total soluble solid, radical scavenging capacities, copper chelating, and ferric reducing activities, without any changes in the concentration of flavonoids [61]. However, the heated apple juice with onion not only exhibited improved values for these parameters but also notably elevated the concentration of flavonoids [61]. It has been suggested that heating markedly elevates the functionality of onion, including polyphenol concentration, antioxidant activity, and metal chelating capacity [60,64]. It appears that heat treatment and onion addition together can serve as a favorable approach to increase the color and nutritional quality of apple juice.

### 3.2. Pineapple

Pineapple is a popular fruit crop worldwide that is consumed fresh or in various processed forms. Pineapple juice is one of the widely consumed pineapple products because of its pleasant aroma and flavor [62]. Interestingly, pineapple juice can be used as an anti-browning agent. Lozano-de-Gonzalez et al. reported that pineapple juice and ion-exchanged pineapple juice were comparable to sulfite, a widely used inhibitor for inhibiting the enzymatic browning of fresh and dried apple rings [63]. When pineapple juice was fractionated using various size and charge separation procedures, all fractions inhibited the enzymatic browning of crude apple extracts by at least 26% [62,63]. The inhibitory effect of pineapple juice on banana has also been reported. When banana slices were treated with pineapple juice at 15 °C for 3 days, pineapple juice significantly inhibited the browning of the banana slices. The effect was comparable to 8 mM ascorbic acid but was less than that of 4 mM sodium metabisulfite [62]. The fractionation of pineapple juice showed that directly eluted fraction suppressed banana PPO by almost 100% as compared to the control group (Table 4). Further analysis of the directly eluted fraction revealed malic and citric acids as the major compounds that can suppress the activity of banana PPO [62].

### 3.3. Lemon, Grape, and Wine

Enzymatic browning also occurs during dough formation and is a serious problem for fresh-pastry products because it affects the dough characteristics, thereby reducing consumer acceptance [21]. A study investigated the effects of lemon juice, grape juice, and white wine on the browning of pastry dough samples. Pastry dough with 5 g of lemon juice added to it, exhibited brighter base color presumably because of the anti-browning effects of citric and ascorbic acids in the lemon juice, but the brightness (L*) decreased as time passed, possibly due to the depletion of the anti-browning compounds [21]. Grape juice contains various polyphenols, but it has shown a low anti-browning potential regardless of the polyphenolic concentration. White wine containing polyphenols, alcohol, and acids also delayed the browning of the dough samples. The anti-browning effect of white wine was better than that of grape juice but lesser than that of lemon juice [21]. The authors believed that the stronger anti-browning potential of lemon juice than that of white wine and grape juice is associated with its stronger chelating effect, as citric and ascorbic acids present in the lemon juice bind to the active center stronger than malic and tartaric acids present in the wine and grape juices. To reinforce the anti-browning effect of these, lemon juice and white wine were combined based on their inhibitory effects on PPO activity and known reports about their active compounds. This combination efficiently prevented the browning of pastry dough over the entire experimental period of 4 weeks (Table 4). It was suggested that the optimal browning inhibition by the combination treatment was based on their active compounds that acted at different reaction points of the browning process.

### 3.4. Dietary and Herbal Compounds

Recent studies showed that maclurin and swertiajaponin present in fruits and herbs are also strong antioxidants with anti-browning activities on potatoes [36,55,56]. Swertiajaponin (5–500 µM) exhibited an antioxidant activity evidenced by markedly suppressed reactive oxygen species (ROS) generation in an in vitro cell culture experiment. When added to potato extract, swertiajaponin significantly increased the antioxidant properties of the potato extract and suppressed enzymatic browning better than ascorbic acid [56]. In an in vitro study, maclurin at 10 μM reduced ROS generation by approximately 80%, whereas vitamin C at 10 μM decreased it by approximately 43%. When added to potato, treatment with maclurin (1 and 10 µM) significantly lowered Sin1-induced ROS and ONOO^−^ in the potato supernatant. The ROS and ONOO^−^ scavenging activities of ascorbic acid at 10 μM appeared to be similar to that of maclurin at 1 μM in the potato supernatant [55]. Maclurin suppressed the enzymatic browning of the potato supernatant for a long period of approximately 5 weeks at 4 °C.

Previous studies have identified strong tyrosinase inhibitory activity in plants of the family Moraceae, especially *Morus alba* and *Ficus auriculata* [65,66]. The active compounds with tyrosinase inhibitory activity include flavones (30%), flavanones (14%), and 2-arylbenzofurans (10%), and their inhibitory effect was comparable to that of kojic acid [65]. Another study investigated the antioxidant and anti-browning potential of 2-arylbenzofurans, including sanggenofuran A, mulberrofuran D2, mulberrofuran D, morusalfuran B, and mulberrofuran H, present in the root barks of *M. alba* Linn [67]. All the compounds exhibited DPPH radical scavenging activity, with an IC_50_ in the range of 11.58–55.73 μM. Of these, mulberrofuran H and morusalfuran B showed strong antioxidant activities (IC_50_: 11.58 ± 0.85 mM and 12.99 ± 0.43 mM, respectively) [67]. Moreover, when the anti-browning properties were tested by the tyrosinase inhibition assay using l-tyrosine and l-DOPA as substrates, mulberrofuran H (IC_50_: 4.45 ± 0.55 μM for l-tyrosine and 19.70 ± 0.54 μM for l-DOPA) exhibited the strongest inhibition, comparable to that of kojic acid (IC_50_: 4.49 ± 0.09 μM for l-tyrosine and 7.08 ± 0.57 μM for l-DOPA). The inhibitory effects of the other compounds were moderate and variable, depending on the substrates [67].

Cyclodextrins are naturally occurring cyclic oligosaccharides derived from starch with 6, 7, or 8 glucose residues linked by α(1–4) glycosidic bonds [68]. The application of cyclodextrins as anti-browning compounds in fruit juices has received considerable attention [69]. Various cyclodextrins have been used to investigate the evolution of the color parameters of different fruit juices, such as pear [70], peach [71], apple [72], and grape [73]; the data showed that cyclodextrins can form complexes with PPO substrates, thereby suppressing their oxidation to quinones and subsequent polymerization to brown pigments [69].

Complex and pure dietary ingredients presented in plants are known to possess multiple bioactive components with health benefits. In addition to functionality, they show anti-browning properties through PPO inhibition. Thus, complex and pure dietary components may be an attractive anti-browning agent for the consumer. Given the sustainability perspective, it is timely to identify new anti-browning agents from food by-products and waste.

## 4. Food by-Products and Waste as Anti-Browning Agents

### 4.1. Unripe Grapes

Grapes, one of the most widely used fruit crops in the world, contain a substantial amount of polyphenols, and their content relies on various factors including the climatic conditions and stage of ripeness [74]. It has been reported that 3 to 6 million tons per year of grape pomace were generated after making wines in the period 2000–2013 (Food and Agricultural Organization 2016) [45]. Thus, numerous efforts have been made to use grape-related by-products and waste for decreasing the alcohol concentration and pH of wines. Furthermore, it has been shown that unripe grapes contain higher amount of polyphenols than that of the ripened ones [74]. A study evaluated the anti-browning and antioxidant properties of the unripe grapes. The unripe berries were gathered during bunch thinning of Barbera and Merlot vineyards [45]. Merlot grapes exhibited the strongest antioxidant, ferric reducing, and anti-browning abilities [45]. The beneficial effects of unripe grapes are probably derived from their flavanol (catechin, epicatechin, epicatechin, gallate, epigallocatechin, and epigallocatechin gallate) and phenolic acid (caffeic, chlorogenic and gallic acids) content. Of these, epigallocatechin gallate was the main phenolic compound observed in the unripe grapes (Table 5) [45].

Another study also investigated the active compounds present in the unripe grape juice and their anti-browning effects [74]. They isolated hydroxycinnamoyl acid and tartaric acid esters from the unripe grape juice using chromatographic techniques. Of the main components, caftaric acid inhibited the tyrosinase activity competitively and the inhibitory effect was better than the related caffeic and chlorogenic acids (Table 5) [74]. Accumulated evidence proved that the unripe grapes and related products are a good source of bioactive compounds, which provide health benefits. Thus, the conversion of these agricultural waste into value-added products will be necessary for the food industry [45].

### 4.2. Sapindaceae (Dimocarpus Longan and Nephelium Lappaceum) Seed and Peel By-Products

A substantial amount of by-products is annually generated from the processing of Sapindaceae fruits, such as longan and rambutan, which account for 24.9–40.7% and 52.9–74.7% of the whole fruit on a fresh weight basis, respectively [79]. Although these by-products contain many functional compounds including ellagic acid, gallic acid, corilagin, and geraniin, they are currently discarded as waste [79].

Previous studies have reported the inhibitory activities of longan peel and seed extracts on tyrosinases. The ethanol extract of longan peel at 100 μg/mL suppressed the tyrosinase activity by approximately 23.6%. It appears that the antioxidant activity of longan peel contributes to the PPO-inhibitory action [76]. The aqueous extract of longan seed at 5 mg/mL also inhibited the tyrosinase activity by more than 60%, and its IC_50_ value was found to be 2.9–3.2 mg/mL (Table 5) [75].

### 4.3. Microwaved Thinned Nectarine Extracts

By-products are also generated during fruit thinning, which is necessary for increasing the size of the remaining fruit, decreasing the hazard of limb breakage, and avoiding an alternate bearing cycle [77]. A substantial amount of effort and cost has been spent on this process [80]. Besides, these thinned fruits include a lot of functional compounds that have diverse health-promoting effects [81]. Nevertheless, thinned fruits are usually abandoned. Thus, the use of these by-products produces an extra profit for the agricultural and food industry. There were efforts underway to elucidate novel ways to utilize thinned fruits as anti-browning agents. A study investigated the potential of the Maillard reaction products generated during the microwave exposure of thinned nectarines to suppress the enzymatic browning reaction catalyzed by PPO [77]. The study reported that the thinned nectarine extracts after the exposure of high microwave power levels (500, 1000, and 1500 W) inhibited PPO derived from mushrooms. It appears that the thinned nectarine extracts act as mixed-type inhibitors of PPO, exhibiting an irreversible inactivation. The authors speculated that the mechanism underlying the extract-mediated PPO inhibition is possibly derived from the Maillard reaction products produced during microwave exposure [77]. This method is also effective when applied to fruit. A solution of 2% thinned nectarine extract exposed to microwave (1500 W) efficiently suppressed enzymatic browning in minimally processed peaches for 8 days of storage (Table 5) [77]. It is necessary for commercial use to further study the optimization of obtaining Maillard reaction products from thinned nectarines and detect the possible formation of mutagenic compounds associated with the Maillard reaction [77].

### 4.4. Tomato Skin

Tomato skin is a low-cost by-product with very high lycopene content and may contain approximately 14-fold higher lycopene content than the internal tissues [82]. In addition, it has a high potential to be incorporated as an antioxidant agent in the anti-browning dipping treatments [78]. However, lycopene is unstable and is degraded under high temperatures and in the presence of O_2_. Encapsulation was shown to decrease these losses while allowing a controlled lycopene release over time [78]. A study successfully used the lycopene microspheres to prevent enzymatic browning and elevate the nutritional quality of the fresh-cut apples. Heat extraction of lycopene combined with TiO_2_ nanoparticles from the tomato skin reaches excellent lycopene extraction yield, with predominance of *cis*-lycopene isomers [78]. When fresh-cut apples were treated with lycopene microspheres, enzymatic browning was prevented for 9 days at 5 °C, without changing the physicochemical or microbial quality (Table 5) [78]. Furthermore, the incorporation of lycopene microspheres strengthened the beneficial properties of apples, showing elevation of phenolic compounds by up to 56% (for chlorogenic acid) [78].

## 5. Conclusions

Development of anti-browning agents in the food industry is critical to maintain the quality of FV products. Traditionally, effectiveness and cost-efficiency are important factors to be considered for developing anti-browning agents. However, current trends in anti-browning agents need to meet consumer needs that demand attention to natural sources, health benefits, and sustainability. The anti-browning properties of food ingredients, such as onion, pineapple, lemon, grape, and wine, and various other dietary components have been studied. Some of them strongly inhibit PPOs and exhibit biological activity. Moreover, there are efforts underway to elucidate the anti-browning activities of food by-products and waste.

## Figures and Tables

**Table 1:** Antioxidant effects of chemical compounds.

Compound	Structure	Conc.^1^	Product	Effect	Ref.
Ascorbic acid	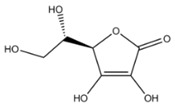	5 mM 0.3 mM	Apple juice	Reducing oxidant substrates Reduction of *o*-quinones to their precursor diphenols	[34,35]
N-acetyl cysteine	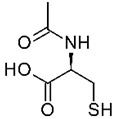	1.7 mM 25 mM	Potato Apple	Competitive inhibition of PPO ^2^ Reactive oxygen species scavenger	[33]
4-Hexylresorcinol	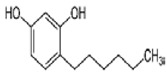	1.8 μM	Peer Apple	PPO inactivation Synergistic inhibition with ascorbic acid and N-acetyl cysteine	[32,33]
Glutathione	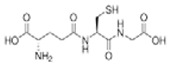	0.08%	Peer Apple juice	Inhibited PPO activity	[36,37]
Cysteine hydrochloride	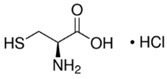	1.8 μM 1%	Fruit salad	Inhibited PPO activity	[38,39]
Erythorbic acid	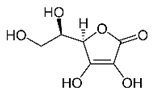	19.6 μM	Fruit salad	Inhibited PPO activity Oxygen scavenger	[38,39]

^1^ Conc.: concentration; ^2^ PPO: polyphenoloxidase.

**Table 2:** Chelating agents and acidulants of chemical compounds.

Compound	Structure	Conc. ^1^	Product	Effect	Ref.
Citric acid	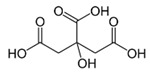	2.7 mM	Lettuce-head	PPO ^2^ noncompetitive inhibitor	[38]
Kojic acid	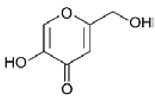	25 μM	Apple Potato	Strong chelator such as Fe(III) and Cu(II) Inactivated PPO enzyme (bind to Cu in PPO)	[42,43]
Oxalic acid	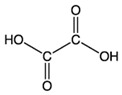	2.0 mM 10 μM	Apple Lettuce	Chelating copper from the active site of PPO PPO noncompetitive inhibitor	[38]
Caffeic acid	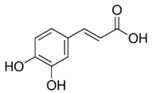	955.7 μM	Apple juice Unripe grapes juice	Low inhibitory activity on enzymatic browning	[44,45]
Chlorogenic acid	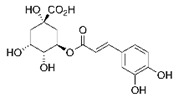	1 mM	Loquat juice	Prevention of enzymatic browning through inactivating PPO	[46]
Coumaric acid	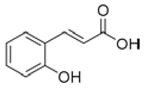	50 μg/mL	Potato Apple puree	Inhibited PPO activity	[47]
Gallic acid	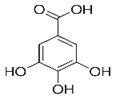	59.2 μM	Unripe grapes juice	Low inhibitory activity on enzymatic browning	[45]
Carboxylic acid	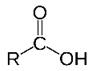	1%	Apple	Inhibitory effects on enzymatic browning due to metal-chelating activities or lowering pH	[43]
Oxaloacetic acid	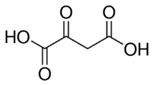	1%	Apple	Inhibitory effects on enzymatic browning due to metal-chelating characteristics or lowering pH	[43]
Lactic acid	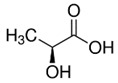	1%	Apple	Inhibitory effects on enzymatic browning because of their metal- chelating characteristics or lowering pH	[43]
Malic acid	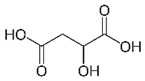	163.8 mM	Unripe grapes juice	Inhibitory effects on enzymatic browning because of their metal-chelating characteristics or lowering pH	[43]
Pyruvic acid	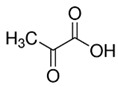	1%	Apple	Inhibitory effects on enzymatic browning due to metal-chelating characteristics or lowering pH	[43]
Acetic acid	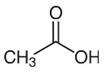	0.1%	Lettuce-head Cabbage	No apparent effect on PPO activity	[48,49]
Succinic acid	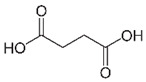	536.7 mM	Unripe grapes juice	Less effective in controlling enzyme browning	[45]
Formic acid	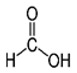	1%	Apple	Less effective in controlling enzymatic browning Inhibitory effects on enzymatic browning because of their metal-chelating characteristics or lowering pH	[43,45]

^1^ Conc.: concentration; ^2^ PPO: polyphenoloxidase.

**Table 3:** Mixed-type inhibitors of chemical compounds.

Compound	Structure	Conc. ^1^	Product	Effect	Ref.
Maclurin	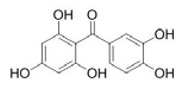	1 and 10 μM	Potato	ROS ^2^ and peroynitrite ^3^ scavenger Tyrosinase binding and inactivation	[57]
Swertiajaponin	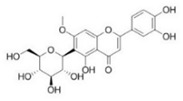	5–500 μM	Potato	Suppressed ROS generation Tyrosinase binding and inactivation	[36,56]

^1^ Conc.: concentration; ^2^ ROS: reactive oxygen species; ^3^ Peroxynitrite-: ONOO^−^.

**Table 4:** Application of natural anti-browning agents on fruits and vegetables.

Source	Extraction Condition	Product	Conc. ^1^	Result	Ref
Onion	Heat (96 °C/1 h)	Apple juice	2.5%	Reduced browning by inhibition of PPO ^2^ (53.87%)	[61]
	Heat (100 °C/10 min)	Potato extract/slice	3.1 mg/mL	Decreased browning by non-competitive inhibition of PPO	[59]
	Heat (100 °C/10 min)	Pear juice	60 mg/mL	Prevention of enzymatic browning by PPO inhibition (45.9%)	[60]
Pine-apple	Crush and freeze dry	Banana slice	Dipping in 12 °Brix	Effective enzymatic browning inhibition in banana slices stored at 15 °C for 3 days (PPO inhibition 52.3%)	[62]
	Concentrated pineapple juices	Apple rings	Dipping in 13.0 °Brix	Inhibition of PPO at least 25%	[63]
Wine	Commercial product	Pastry dough 10 gdm		Prevention of enzymatic browning and mold formation	[21]
Lemon	Freshly squeezed				

^1^ Conc.: concentration; ^2^ PPO: polyphenoloxidase.

**Table 5:** Food by-products and waste with anti-browning agents.

Source	Extraction Condition	Product	Result	Ref
Unripe grape	Crushing and vacuum filtration components (Separated by HPLC)	Caftaric acid	Inhibition of tyrosinase competitively (Tyr IC_50_^1^: 30 µM caftaric acid, 42 µM caffeic acid and 65 µM chlorogenic acid)	[74]
		Chlorogenic acid		
		Caffeic acid		
	Centrifuged and filtration	Merlot and Barbera in the 2013 and 2014 seasons	Antioxidant and whitening activities (Tyr IC_50_: 14.7 mmol/L M1, 16.8 mmol/L M2, 2.5 mmol/L B1, and 3.2 mmol/L B2) 2013: M1, B1 and 2014: M2, B2)	[45]
Longan	Dry and ultra-high-pressure-assisted extraction (UHPE)	100 g/mL UHPE (pressures of 500 MPa)	High phenolic contents, high antioxidant and anti-tyrosinase activities (anti-tyrosinase activity: 23.6 ± 1.2)	[75]
	Extracted and lyophilized	Dried seed extracts	High antioxidant activity and tyrosinase inactivation (Tyr IC_50_ values: 2.9 and 3.2 mg/mL)	[76]
Thinned nectarine extracts	After dried nectarines were mixed with distilled water, microwaved	Microwave (1500 W power)-treated thinned nectarine extracts	1500 W MRP inhibited the enzymatic browning in minimally processed peaches for 8 days of storage	[77]
Tomato skin	High lycopene extraction from tomato skin	Dipping solution containing 2 g of lycopene microspheres per L. (in fresh-cut processing of apples)	Reduced browning and some bioactive compounds even enhanced for 9 days Browning index (BI = 43.8)	[78]

^1^ Tyr IC_50_: 50% inhibitory concentration of tyrosinase activity.
